# Characteristics of Plausible Source Cases Responsible for Recent *Mycobacterium tuberculosis* Transmission, United States, 2018–2022

**DOI:** 10.3201/eid3206.260104

**Published:** 2026-06

**Authors:** Steve Kammerer, Daniel Flanagan, Kala Raz, Tambi Shaw, Jonathan Wortham, Sarah Talarico

**Affiliations:** Centers for Disease Control and Prevention, Atlanta, Georgia, USA (S. Kammerer, K. Raz, J. Wortham, S. Talarico); Emory University, Atlanta (D. Flanagan); California Department of Public Health, Richmond, California, USA (T. Shaw)

**Keywords:** Tuberculosis and other mycobacteria, Mycobacterium tuberculosis, bacteria, TB, respiratory infections, disease transmission, infectious, molecular epidemiology, whole-genome sequencing, public health surveillance, social determinants of health, machine learning, United States

## Abstract

Tuberculosis (TB) outbreaks in the United States can cause substantial illness. Using surveillance and genotyping data, we applied a plausible source–case algorithm to identify TB cases reported during 2018–2020 responsible for secondary cases attributed to recent *Mycobacterium tuberculosis* transmission during 2020–2022. We used mixed models and a machine learning workflow to assess sociodemographic, clinical, and social risk factors associated with plausible sources. In mixed models, sputum smear positivity, cavitary disease, race/ethnicity other than non-Hispanic White or non-Hispanic Asian, age <65 years, US birth, and homelessness were associated with plausible sources. An adaptive boosting model achieved an area under the receiver operating characteristic curve of 0.81 on test data. Transmission was heterogeneous; 8.1% of sources linked to 3–15 secondary cases accounted for 24.9% of transmission events. Focusing case management and contact investigations on cases with the characteristics we identified could reduce *M. tuberculosis* transmission and improve TB prevention.

Tuberculosis (TB) is the leading infectious cause of death worldwide (*1*). The World Health Organization estimated that >10 million persons had TB develop during 2023 (*1*). TB incidence in the United States is low at <3 cases/100,000 population (≈9,000 annual cases) reported in recent years (*2*). Most US TB cases reflect reactivation of past *Mycobacterium tuberculosis* infection rather than recent transmission within US borders and most cases are diagnosed among non–US-born persons (*2*,*3*). However, outbreaks resulting from *M. tuberculosis* transmission within the United States continue to occur, disproportionately affect US-born persons, and can cause substantial illness (*4**–**10*). Therefore, targeted interventions to prevent *M. tuberculosis* transmission and TB outbreaks are crucial.

Public health interventions to prevent reactivation of latent TB differ from interventions designed to prevent *M. tuberculosis* transmission (*11*). Whereas preventing reactivation requires diagnosing and treating asymptomatic latent TB among persons with epidemiologic risk factors (e.g., birth outside the United States) (*3*,*12*), preventing *M. tuberculosis* transmission requires intensive case management to promptly initiate treatment to cure TB and identify, fully evaluate, and treat close contacts of TB case-patients (*13*). Clinically distinguishing between TB resulting from reactivation versus recent *M. tuberculosis* transmission often is impossible; thus, estimates of the percentage of cases attributable to recent transmission are needed to inform prioritization of resources and activities for TB prevention (*14**–**20*). With this aim, the Centers for Disease Control and Prevention (CDC) developed and validated a plausible source–case algorithm to estimate the percentage of TB cases attributable to recent transmission within US borders over a 2-year period (*17*,*18*). On the basis of that algorithm, ≈10%–15% of TB cases nationwide were attributed to recent *M. tuberculosis* transmission, but local estimates varied widely (*17*,*21*). During 2020–2021, a total of 1,400 cases were attributed to recent transmission in the 50 US states and the District of Columbia (*21*). Recent literature also suggests heterogeneity in transmission at the individual case level; some analyses have proposed that transmission associated with a few TB cases accounts for a disproportionate share of secondary cases (*15*,*16*,*22**–**25*) and that transmission associated with those cases is more likely to result in outbreaks (*15*,*24*). 

Focused interventions to prevent transmission, such as intensive case management and contact investigations for cases most likely to generate secondary cases, could reduce TB illnesses. Nonetheless, few analyses have sought to describe the characteristics of cases presumed to have transmitted *M. tuberculosis* to secondary cases (*17*,*18*). We used national molecular and case surveillance data to characterize the sociodemographic, clinical, and social risk factors associated with plausible TB source cases in the United States.

## Materials and Methods

### Data Sources

We obtained patient and case characteristics from the National Tuberculosis Surveillance System for incident TB cases reported to CDC during 2018–2022; demographic, clinical, and social risk factors are reported for each case (*21*). We included cases from all 50 US states and the District of Columbia.

For community-level characteristics, we used the CDC/Agency for Toxic Substances and Disease Registry’s Social Vulnerability Index (SVI) based on the US Census Bureau’s 2016–2020 American Community Survey (*26*). The SVI provides a relative vulnerability ranking for each US county by using 16 social factors grouped into 4 themes (i.e., socioeconomic status [SES], household characteristics, racial and ethnic minority status, and housing type and transportation) and an overall ranking. Rankings are values from 0 to 1, where higher values indicate higher social vulnerability.

We used genotyping data generated from whole-genome sequencing methods that assign a whole-genome multilocus sequence type (wgMLSType). *M. tuberculosis* isolate sequencing has been routinely available for >96% of culture-confirmed TB cases reported since 2018. The wgMLSType is assigned by comparing the sequences of 2,672 genetic loci and designating the same wgMLSType to cases that have matching patterns at >99.7% of loci.

We performed whole-genome single-nucleotide polymorphism (wgSNP) comparisons for all isolate pairs identified by the plausible source–case algorithm as a potential source and secondary case during the study period. We used BioNumerics 7.6.3 (Applied Maths, http://www.applied-maths.com) to perform wgSNP comparisons, which provide increased molecular resolution compared with wgMLSType. We excluded any single-nucleotide polymorphisms (SNPs) in a wgSNP comparison if total coverage was <5 reads or if it contained any ambiguous or unreliable bases or gaps. We also excluded all SNPs that were <12 bp from another SNP.

### Recent Transmission Estimates

We applied the field-validated plausible source–case algorithm to culture-confirmed, genotyped TB cases reported during 2020–2022 that had nonunique wgMLSTypes. We attributed a case to recent transmission if >1 plausible source case of pulmonary or laryngeal TB was diagnosed within the previous 2 years in persons >10 years of age who had matching wgMLSType and resided within a 10-mile radius (*18*).

We excluded plausible source cases with isolates that differed by >5 SNPs from the secondary case’s isolate to restrict the dataset to case pairs that are most likely to represent recent transmission (*27*). We extracted all remaining plausible source cases reported during 2018–2020; we classified any case not identified as a plausible source case for >1 cases attributed to recent transmission as a nonplausible source. Limiting the plausible source–case population to 2018–2020 enabled an equal 2-year follow-up for any subsequent secondary cases when assigning plausible source case status.

### Study Population

We used a single binary outcome for analyses, defined as whether a case was identified as a plausible source case during 2018–2020 for >1 secondary cases attributed to recent transmission during 2020–2022. Because a secondary case can have multiple plausible sources, we assessed 2 scenarios: inclusion of all plausible sources (all scenario) and selection of a single most likely plausible source (most likely scenario).

**Figure 1:**
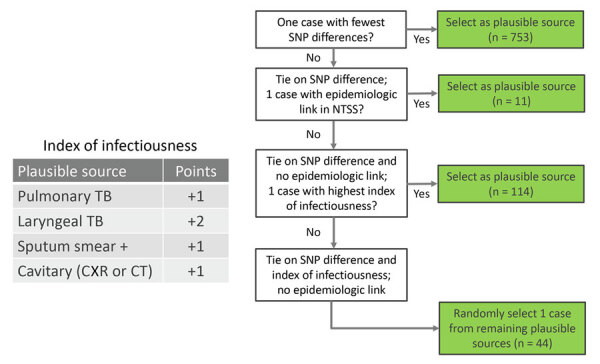
Determination of the most likely plausible source cases responsible for recent *Mycobacterium tuberculosis* transmission, United States, 2018–2022. Flowchart shows the structured decision process used to identify the most likely plausible source case. The algorithm evaluated SNP differences, epidemiologic links in the NTSS, and an index of infectiousness. If ties remained after applying those criteria, 1 plausible source case was randomly selected from the remaining candidates. CXR, chest radiograph; CT, computed tomography; NTSS, National Tuberculosis Surveillance System; SNP, single-nucleotide polymorphism; TB, tuberculosis.

We developed a decision tree to determine the most likely plausible source case. For each secondary case, we selected a single most likely source by prioritizing minimum wgSNP distance, then documented epidemiologic link, followed by highest infectiousness index. We resolved ties at random (Figure 1). We applied the most likely scenario to plot the distribution of the number of secondary cases attributed to recent transmission during 2020–2022 associated with each plausible source identified during 2018–2020.

### Descriptive Analyses

We compared individual-level demographic, clinical, and social risk factors and community-level SVI measures of plausible versus nonplausible sources under both scenarios. To assess characteristics associated with plausible source cases that transmit to >3 secondary cases, we performed an additional descriptive analysis by using the most likely scenario and stratified the outcome into 0, 1–2, and >3 secondary cases. We used χ^2^ test of independence or Fisher exact test for categorical variables and Wilcoxon rank-sum tests for continuous variables. We considered p<0.05 statistically significant.

### Generalized Linear Mixed Models

We fit a multivariable generalized linear mixed model (GLMM) to determine individual- and community-level characteristics associated with being a plausible source case and developed separate models for the all and most likely scenarios. We incorporated a random intercept for county to accommodate clustering of cases by county and inclusion of county-level SVI measures; we assessed the separate factors in any SVI theme that were significantly associated with the outcome. We performed backward elimination starting with all variables included in the descriptive analysis. We assessed effect modification in the reduced model and evaluated multicollinearity by using variance inflation factors. We sequentially removed covariates if their exclusion decreased the Akaike information criterion, finalized models when no further improvement was observed, and calculated adjusted odds ratios (aORs) and 95% CIs.

### Machine Learning Models

We also evaluated machine learning models (MLMs) that predict whether a TB case is estimated to be a plausible source. Using the most likely scenario, we developed a machine learning workflow to assess 10 different methods and included all features (i.e., variables) from the descriptive analysis (*28*) (Appendix).

### Sensitivity Analyses

We reran both the GLMM and MLM workflows to evaluate the effects of using the infectiousness index to determine the outcome and smear positivity and cavitary disease as predictors. First, we restricted the data to the subset of secondary cases for which the most likely plausible source was determined using only wgSNP difference or epidemiologic link. Then, we reran the selection hierarchy without the infectiousness index (Appendix).

We used SAS version 9.4 (SAS Institute, Inc., https://www.sas.com) for data management, descriptive analyses, and GLMM development and Python version 3.9.13 (Python Software Foundation, https://www.python.org) for MLM analyses. CDC determined this activity to be routine public health surveillance and not human subjects research. WGS was performed as part of routine public health surveillance and no new sequence data were generated as part of this study. Sequence data included in this analysis are available in the National Center for Biotechnology Information (BioProject no. PRJNA1237251).

## Results

### Study Population

**Figure 2:**
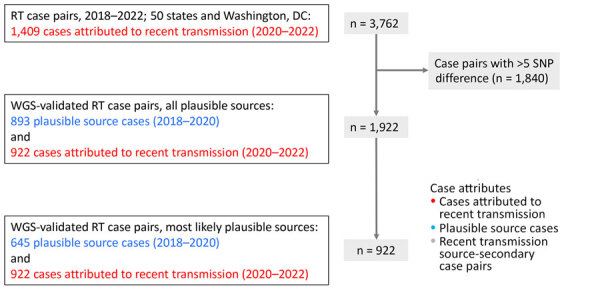
Flow diagram showing selection of recent transmission source–secondary case pairs from 50 states and Washington, DC, included in an analysis of plausible source cases responsible for recent *Mycobacterium tuberculosis* transmission, United States, 2018–2022. Among 3,762 RT case pairs identified, case pairs with >5 SNP differences were excluded (n = 1,840), leaving 1,922 WGS–validated RT case pairs. The analytic dataset included 922 TB cases attributed to RT during 2020–2022 and plausible source cases identified during 2018–2020 (all plausible source cases [n = 893] and most likely plausible source cases [n = 645]). RT, recent transmission; SNP, single-nucleotide polymorphism; TB, tuberculosis; WGS, whole-genome sequencing.

During 2018–2022, a total of 41,264 TB cases were reported from the 50 US states and the District of Columbia, of which 32,110 (77.8%) were culture-confirmed and genotyped, and 61% (n = 19,577) of culture-confirmed and genotyped cases were reported during 2018–2020. Using the plausible source–case algorithm, we identified 3,762 recent transmission source–secondary case pairs for which 1,922 (51.1%) were supported by wgSNP analysis (Figure 2). The 1,922 case pairs comprised 922 cases attributed to recent transmission during 2020–2022, indicating a mean of 2.1 (range 1–24) plausible source cases per secondary case. We identified 893 (4.6%) unique plausible source cases during 2018–2020 for the all scenario (Table 1; Figure 2; Appendix Table 1). We found secondary cases attributed to recent transmission in 44 states.

**Table 1:** Characteristics of all plausible source TB cases during 2018–2020 responsible for recent *Mycobacterium tuberculosis* transmission during 2020–2022, United States*

Characteristic	Nonplausible source	Plausible source	p value†
Total cases, n = 19,577	18,684 (95.4)	893 (4.6)	
Sex			<0.001
M	11,465 (61.4)	631 (70.7)	
F	7,216 (38.6)	262 (29.3)	
Unknown	3 (0)	0	
Age, y			<0.001
<5	185 (1.0)	0	
5–14	191 (1.0)	13 (1.5)	
15–24	1,802 (9.6)	120 (13.4)	
25–44	5,521 (29.5)	338 (37.9)	
45–64	5,503 (29.5)	337 (37.7)	
>65	5,482 (29.3)	85 (9.5)	
Origin of birth			<0.001
United States	4,829 (25.8)	536 (60.0)	
Non-US country	13,826 (74.0)	356 (39.9)	
Unknown	29 (0.2)	1 (0.1)	
Race and ethnicity‡			<0.001
Hispanic or Latino	5,459 (29.2)	290 (32.5)	
American Indian/Alaska Native	161 (0.9)	47 (5.3)	
Native Hawaiian/Pacific Islander	215 (1.2)	29 (3.2)	
Black	3,377 (18.1)	325 (36.4)	
Asian	7,161 (38.3)	86 (9.6)	
White	2,115 (11.3)	104 (11.6)	
Multiple race	137 (0.7)	6 (0.7)	
Unknown	59 (0.3)	6 (0.7)	
Resident of a correctional facility at TB diagnosis			<0.001
Y	464 (2.5)	49 (5.5)	
N	18,154 (97.2)	834 (93.4)	
Unknown	66 (0.3)	10 (1.1)	
Experiencing homelessness within past 12 mo			<0.001
Y	744 (4.0)	138 (15.4)	
N	17,776 (95.1)	740 (82.9)	
Unknown	164 (0.9)	15 (1.7)	
Excess alcohol use within past 12 mo			<0.001
Y	1,618 (8.7)	184 (20.6)	
N	16,783 (89.8)	687 (76.9)	
Unknown	283 (1.5)	22 (2.5)	
Noninjection drug use within past 12 mo			<0.001
Y	1,213 (6.5)	204 (22.8)	
N	17,022 (91.1)	650 (72.8)	
Unknown	449 (2.4)	39 (4.4)	
Sputum smear			<0.001
Positive	8,788 (47.0)	621 (69.5)	
Negative	7,759 (41.5)	217 (24.3)	
Not done	2,119 (11.3)	55 (6.2)	
Unknown	18 (0.1)	0	
Cavitary disease§			<0.001
Y	6,638 (35.5)	541 (60.6)	
N	9,877 (52.9)	310 (34.7)	
Unknown	2,169 (11.6)	42 (4.7)	
Contact of infectious tuberculosis patient during past 2 y			<0.001
Y	970 (5.2)	122 (13.7)	
Unknown/not reported	17,714 (94.8)	771 (86.3)	
Median Social Vulnerability Index score (IQR) ¶	0.73 (0.49–0.86)	0.83 (0.66–0.89)	<0.001#
Socioeconomic status	0.65 (0.34–0.86)	0.80 (0.58–0.92)	<0.001#
Household characteristics	0.45 (0.23–0.70)	0.56 (0.32–0.74)	<0.001#
Racial and ethnic minority status	0.91 (0.78–0.96)	0.92 (0.82–0.97)	<0.001#
Housing type and transportation	0.78 (0.61–0.90)	0.83 (0.68–0.91)	<0.001#

*Values are no. (%) except as indicated. IQR, interquartile range; TB, tuberculosis. †χ^2^ test or Fisher exact test when <5 cases expected. ‡Except for Hispanic or Latino, all are non-Hispanic. §Evidence of >1 lung cavities with chest radiograph, chest computerized tomography, or both. ¶Overall Social Vulnerability Index includes 16 US Census indicators from the 5-year American Community Survey grouped into 4 themes: socioeconomic status, household characteristics, racial and ethnic minority status, and housing type and transportation. Ranking values range from 0 to 1, and higher values indicate higher vulnerability and are merged to each case using the patient’s county of residence. #Wilcoxon rank sum test.

For the most likely scenario, we identified 645 (3.3%) unique plausible source cases during 2018–2020 (Table 2; Appendix Table 2). Among the 922 cases attributed to recent transmission, we chose 753 (81.7%) plausible source cases by using wgSNP difference, 114 (12.4%) by using the index of infectiousness, 44 (4.8%) by using random selection, and 11 (1.2%) by using epidemiologic links (Appendix Table 4).

**Table 2:** Characteristics of most likely plausible source TB cases during 2018–2020 responsible for recent *Mycobacterium tuberculosis* transmission during 2020–2022, United States*

Characteristic	Nonplausible source	Plausible source	p value†
Total no. (%) cases, n = 19,577	18,932 (96.7)	645 (3.3)	
Sex			<0.001
M	11,640 (61.5)	456 (70.7)	
F	7,289 (38.5)	189 (29.3)	
Unknown	3 (0.0)	0 (0.0)	
Age, y			<0.001
<5	185 (1.0)	0 (0.0)	
5–14	198 (1.0)	6 (0.9)	
15–24	1,828 (9.7)	94 (14.6)	
25–44	5,620 (29.7)	239 (37.1)	
45–64	5,597 (29.6)	243 (37.7)	
>65	5,504 (29.1)	63 (9.8)	
Origin of birth			<0.001
United States	5,022 (26.5)	343 (53.2)	
Non-US country	13,881 (73.3)	301(46.7)	
Unknown	29 (0.2)	1 (0.2)	
Race and ethnicity‡			<0.001
Hispanic or Latino	5,513 (29.1)	236 (36.6)	
American Indian/Alaska Native	186 (1.0)	22 (3.4)	
Native Hawaiian/Pacific Islander	221 (1.2)	23 (3.6)	
Black	3,486 (18.4)	216 (33.5)	
Asian	7,175 (37.9)	72 (11.2)	
White	2,150 (11.4)	69 (10.7)	
Multiple race	141 (0.7)	2 (0.3)	
Unknown	60 (0.3)	5 (0.8)	
Resident of a correctional facility at time of TB diagnosis			<0.001
Y	483 (2.6)	30 (4.7)	
N	18,381 (97.1)	607 (94.1)	
Unknown	68 (0.4)	8 (1.2)	
Experiencing homelessness within past 12 mo			<0.001
Y	793 (4.2)	89 (13.8)	
N	17,973 (94.9)	543 (84.2)	
Unknown	166 (0.9)	13 (2.0)	
Excess alcohol use within past 12 mo			<0.001
Y	1,683 (8.9)	119 (18.5)	
N	16,961 (89.6)	509 (78.9)	
Unknown	288 (1.5)	17 (2.6)	
Noninjection drug use within past 12 mo			<0.001
Y	1,273 (6.7)	144 (22.3)	
N	17,199 (90.9)	473 (73.3)	
Unknown	460 (2.4)	28 (4.3)	
Sputum smear			<0.001
Positive	8,909 (47.1)	500 (77.5)	
Negative	7,863 (41.5)	113 (17.5)	
Not done	2,142 (11.3)	32 (5.0)	
Unknown	18 (0.1)	0 (0.0)	
Cavitary disease§			<0.001
Y	6,737 (35.6)	442 (68.5)	
N	10,004 (52.8)	183 (28.4)	
Unknown	2,191 (11.6)	20 (3.1)	
Contact of infectious tuberculosis patient during prior 2 y			<0.001
Y	1,030 (5.4)	62 (9.6)	
Unknown/not reported	17,902 (94.6)	583 (90.4)	
Median Social Vulnerability Index score (IQR)¶	0.73 (0.49–0.86)	0.80 (0.65–0.89)	<0.001#
Socioeconomic status	0.65 (0.34–0.87)	0.79 (0.56–0.90)	<0.001#
Household characteristics	0.46 (0.23–0.70)	0.55 (0.32–0.74)	<0.001#
Racial and ethnic minority status	0.91 (0.78–0.97)	0.91 (0.81–0.97)	0.006#
Housing type and transportation	0.78 (0.61–0.90)	0.82 (0.67–0.91)	<0.001#

*Values are no. (%) except as indicated. IQR, interquartile range; TB, tuberculosis. †χ^2^ test or Fisher exact test when <5 cases expected. ‡Except for Hispanic or Latino, all are non-Hispanic. §Evidence of >1 lung cavities with chest radiograph, chest computerized tomography, or both. ¶Overall Social Vulnerability Index includes 16 US Census indicators from the 5-year American Community Survey grouped into 4 themes: socioeconomic status, household characteristics, racial and ethnic minority status, and housing type and transportation. Ranking values range from 0 to 1, and higher values indicate higher vulnerability and are merged to each case using the patient’s county of residence. #Wilcoxon rank sum test.

### Descriptive Analyses

In the all scenario, plausible sources were more often male sex, US-born, <65 years of age, and other than non-Hispanic White or non-Hispanic Asian race/ethnicity, and they more frequently reported homelessness and substance use. Those plausible sources also more often had smear positivity and cavitary disease. County-level social vulnerability was higher among plausible sources, especially the SES theme (Table 1; Appendix Table 1).

Using the most likely scenario, characteristic distribution differed greatly across the 3 transmission categories (nonplausible source, plausible source for 1–2 cases, and plausible source for >3 cases) (Table 3; Appendix Table 3). Descriptively, among plausible source cases estimated to have transmitted to >3 (range 3–15) secondary cases, the highest percentages were among persons reporting male sex, 25–44 years of age, US-born, non-Hispanic Black race/ethnicity, experiencing homelessness, noninjection drug use, sputum smear positivity, and cavitary disease. Social vulnerability, including all 4 SVI themes, was also highest for the >3 secondary cases category.

**Table 3:** Characteristics of most likely plausible TB cases during 2018–2020 responsible for recent *Mycobacterium tuberculosis* transmission to >1 secondary TB cases during 2020–2022, United States*

Characteristic	Nonplausible source	Plausible source for >1 TB cases		p value†
		1–2 cases	>3 cases	
Total no. (%) cases, n = 19,577	18,932 (96.7)	593 (3.0)	52 (0.3)	
Sex				<0.001
M	11,640 (61.5)	415 (70.0)	41 (78.9)	
F	7,289 (38.5)	178 (30.0)	11 (21.1)	
Unknown	3 (0.0)	0 (0.0)	0 (0.0)	
Age, y				<0.001
<5	189 (1.0)	0 (0.0)	0 (0.0)	
5–14	198 (1.0)	6 (1.0)	0 (0.0)	
15–24	1,828 (9.7)	87 (14.7)	7 (13.5)	
25–44	5,620 (29.7)	214 (36.1)	25 (48.1)	
45–64	5,597 (29.6)	225 (37.9)	18 (34.6)	
>65	5,504 (29.1)	61 (10.3)	2 (3.8)	
Origin of birth				<0.001
United States	5,022 (26.5)	306 (51.6)	37 (71.2)	
Non-US country	13,881 (73.3)	286 (48.2)	15 (28.8)	
Unknown	29 (0.2)	1 (0.2)	0 (0.0)	
Race and ethnicity‡				<0.001
Hispanic or Latino	5,513 (29.1)	227 (38.3)	9 (17.3)	
American Indian/Alaska Native	186 (1.0)	15 (2.5)	7 (13.5)	
Native Hawaiian/Pacific Islander	221 (1.2)	22 (3.7)	1 (1.9)	
Black	3,486 (18.4)	188 (31.7)	28 (53.8)	
Asian	7,175 (37.9)	68 (11.5)	4 (7.7)	
White	2,150 (11.4)	66 (11.1)	3 (5.8)	
Multiple race	141 (0.7)	2 (0.3)	0 (0.0)	
Unknown	60 (0.3)	5 (0.8)	0 (0.0)	
Resident of a correctional facility at time of TB diagnosis				<0.001
Y	483 (2.5)	27 (4.5)	3 (5.8)	
N	18,381 (97.1)	559 (94.3)	48 (92.3)	
Unknown	68 (0.4)	7 (1.2)	1 (1.9)	
Experiencing homelessness within past 12 mo				<0.001
Y	793 (4.2)	78 (13.1)	11 (21.1)	
N	17,973 (94.9)	504 (85.0)	39 (75.0)	
Unknown	166 (0.9)	11 (1.9)	2 (3.9)	
Excess alcohol use within past 12 mo				<0.001
Y	1,683 (8.7)	109 (18.4)	10 (19.2)	
N	16,961 (89.6)	471 (79.4)	38 (73.1)	
Unknown	288 (1.5)	13 (2.2)	4 (7.7)	
Noninjection drug use within past 12 mo				<0.001
Y	1,273 (6.7)	124 (20.9)	20 (38.5)	
N	17,199 (90.9)	446 (75.2)	27 (51.9)	
Unknown	460 (2.4)	23 (3.9)	5 (9.6)	
Sputum smear				<0.001
Positive	8,909 (47.1)	455 (76.7)	45 (86.5)	
Negative	7,863 (41.5)	108 (18.2)	5 (9.6)	
Not done	2,142 (11.3)	30 (5.1)	2 (3.9)	
Unknown	18 (0.1)	0 (0.0)	0 (0.0)	
Cavitary disease§				<0.001
Y	6,737 (35.6)	402 (67.8)	40 (76.9)	
N	10,004 (52.8)	173 (29.2)	10 (19.2)	
Unknown	2,191 (11.6)	18 (3.0)	2 (3.9)	
Contact of infectious tuberculosis patient during prior 2 y				<0.001
Y	1,030 (5.4)	55 (9.3)	7 (13.5)	
Unknown/not reported	17,902 (94.6)	538 (90.7)	45 (86.5)	
Median Social Vulnerability Index score (IQR)¶	0.73 (0.49–0.86)	0.80 (0.64–0.89)	0.86 (0.78–0.89)	<0.001
Socioeconomic status	0.65 (0.34–0.86)	0.77 (0.56–0.88)	0.84 (0.70–0.92)	<0.001
Household characteristics	0.46 (0.23–0.70)	0.54 (0.31–0.74)	0.57 (0.32–0.77)	<0.001
Racial and ethnic minority status	0.91 (0.78–0.97)	0.91 (0.81–0.97)	0.93 (0.83–0.97)	0.01
Housing type and transportation	0.78 (0.61–0.90)	0.82 (0.67–0.91)	0.85 (0.68–0.91)	<0.001

*Values are no. (%) except as indicated. IQR, interquartile range; TB, tuberculosis. †χ^2^ test or Fisher exact test when <5 cases expected; indicates differences across the 3 categories. ‡Except for Hispanic or Latino, all are non-Hispanic. §Evidence of >1 lung cavities with chest radiograph, chest computerized tomography, or both. ¶Overall Social Vulnerability Index includes 16 US Census indicators from the 5-year American Community Survey grouped into 4 themes: socioeconomic status, household characteristics, racial and ethnic minority status, and housing type and transportation. Ranking values range from 0 to 1, and higher values indicate higher vulnerability and are merged to each case using the patient’s county of residence. p value calculated by Kruskal-Wallis test.

### GLMMs

Of the 19,577 cases available for analysis, we excluded 311 (1.6%) with missing data for sex, birth country, race/ethnicity, sputum smear, or county. We excluded all cases among children 0–4 years of age from analysis because plausible source cases had to be >10 years of age. For the all scenario, we found that the following characteristics were most associated with being a plausible source case: race/ethnicity, specifically Native Hawaiian/Pacific Islander non-Hispanic (aOR 5.34 [95% CI 3.11–9.17]), American Indian/Alaska Native non-Hispanic (aOR 2.12 [95% CI 1.10–4.06]), and Black non-Hispanic (aOR 1.82 [95% CI 1.40–2.36]), compared with White non-Hispanic persons; age <65 years compared with >65 years of age, the greatest association of which was 15–24 years of age (aOR 3.01 [95% CI 2.21–4.30]); US-born (aOR 2.41 [95% CI 1.99–2.91]); experiencing homelessness (aOR 1.89 [95% CI 1.49–2.39]); and indicators of infectiousness, specifically positive sputum smear (aOR 1.71 [95% CI 1.42–2.07]) and cavitary disease (aOR 1.69 [95% CI 1.42–2.00]) (Table 4). Noninjection drug use, male sex, and Hispanic ethnicity also were associated with plausible source cases.

**Table 4:** Multivariable model results for all plausible source TB cases during 2018–2020 responsible for recent *Mycobacterium tuberculosis* transmission during 2020–2022, United States*

Characteristic	Plausible sources, no. (%)	aOR (95% CI)
Total no. (%) cases, n = 19,266	886 (4.6)†	NA
Sex		
M	625 (70.5)	1.26 (1.07–1.48)
F	261 (29.5)	Referent
Age, y		
10–14	13 (1.5)	2.26 (1.17–4.10)
15–24	120 (13.5)	3.01 (2.21–4.30)
25–44	334 (37.7)	2.70 (2.08–3.51)
45–64	334 (37.7)	2.51 (1.94–3.25)
>65	85 (9.6)	Referent
Origin of birth		
United States	535 (60.4)	2.41 (1.99–2.91)
Outside United States	351 (39.6)	Referent
Unknown		
Race and ethnicity‡		
Hispanic or Latino	289 (32.6)	1.52 (1.15–2.02)
American Indian/Alaska Native	47 (5.3)	2.12 (1.10–4.06)
Native Hawaiian/Pacific Islander	29 (3.3)	5.34 (3.11–9.17)
Black	325 (36.7)	1.82 (1.40–2.36)
Asian	86 (9.7)	0.63 (0.45–0.90)
White	104 (11.7)	Referent
Multiple race	6 (0.7)	1.03 (0.41–2.61)
Experiencing homelessness within past 12 mo		
Y	137 (15.5)	1.89 (1.49–2.39)
N	735 (83.0)	Referent
Unknown	14 (1.6)	1.22 (0.62–2.39)
Noninjection drug use within past 12 mo		
Y	204 (23.0)	1.38 (1.12–1.69)
N	646 (72.9)	Referent
Unknown	36 (4.1)	1.68 (1.08–2.59)
Sputum smear		
Positive	615 (69.4)	1.71 (1.42–2.07)
Negative	216 (24.4)	Referent
Not done	55 (6.2)	0.98 (0.69–1.39)
Cavitary disease§		
Y	536 (60.5)	1.69 (1.42–2.00)
N	309 (34.9)	Referent
Unknown	41 (4.6)	0.63 (0.44–0.90)
Type of therapy		
Directly observed therapy alone	591 (66.7)	0.72 (0.51–1.02)
Self-administered therapy alone	9 (1.0)	0.35 (0.16–0.73)
Both	230 (26.0)	0.62 (0.43–0.90)
Unknown	56 (6.3)	Referent
Social Vulnerability Index score¶		
SES—housing cost burden	NA	1.20 (1.06–1.36)
SES—no health insurance	NA	1.13 (1.02–1.25)

*Outcome of plausible source versus not a plausible source for all cases of tuberculosis with a genotyped isolate. Excludes cases with missing sex, origin of birth, race/ethnicity, unknown sputum smear status, 0–4 years of age, and county of residence. All plausible source cases identified by the recent transmission algorithm for a given secondary case are included. aOR, adjusted odds ratio; SES, socioeconomic status; TB, tuberculosis. †Of 19,266 included case-patients. ‡Except for Hispanic or Latino, all are non-Hispanic. §Evidence of >1 lung cavities with chest radiograph, chest computerized tomography, or both. ¶Social Vulnerability Index includes 16 US Census indicators from the 5-year American Community Survey grouped into 4 themes: SES, household characteristics, racial and ethnic minority status, and housing type and transportation. Ranking values range from 0 to 1, and higher values indicate higher vulnerability and are merged to each case using the patient’s county of residence. ORs based on a 0.2-unit (20%) change for that indicator.

Among the SVI themes, only SES was significantly associated with plausible source cases in multivariable generalized linear mixed modeling (p<0.001). Thus, we included 2 of the SES component factors in the final model: housing cost burden (aOR 1.20 [95% CI 1.06–1.36]), defined as households spending >30% of annual income on housing (*26*); and not having health insurance (aOR 1.13 [95% CI 1.02–1.25]). We calculated SVI aORs on the basis of a 0.20-unit (i.e., 20%) increase for each factor. Multicollinearity was modest for age and race/ethnicity, but no variance inflation factors exceeded 4. We found no statistically significant effect modification in the final model. 

The final model for the most likely scenario included the same variables as the all scenario except for SES factors (Table 5). We only retained the not having health insurance (aOR 1.12 [95% CI 1.04–1.21]) factor in that model.

**Table 5:** Multivariable model results for most likely plausible source TB cases during 2018–2020 responsible for recent *Mycobacterium tuberculosis* transmission during 2020–2022, United States

Characteristics	Plausible sources, no. (%)	aOR (95% CI)
Total no. cases, (%); n = 19,126	637 (3.3)	NA
Sex		
M	449 (70.5)	1.26 (1.05–1.52)
F	188 (29.5)	Referent
Age, y		
10–14	6 (0.9)	2.00 (0.83–4.82)
15–24	92 (14.4)	3.03 (2.15–4.27)
25–44	236 (37.1)	2.56 (1.90–3.45)
45–64	240 (37.7)	2.38 (1.78–3.19)
>65	63 (9.9)	Referent
Origin of birth		
United States	341 (53.5)	1.95 (1.58–2.41)
Outside United States	296 (46.5)	Referent
Race and ethnicity†		
Hispanic or Latino	235 (36.9)	1.72 (1.25–2.35)
American Indian/Alaska Native	22 (3.5)	2.71 (1.47–4.98)
Native Hawaiian/Pacific Islander	23 (3.6)	5.72 (3.30–9.93)
Black	216 (33.9)	1.90 (1.42–2.55)
Asian	72 (11.3)	0.76 (0.52–1.11)
White	69 (10.8)	Referent
Experiencing homelessness within past 12 mo.		
Y	88 (13.8)	1.59 (1.22–2.07)
N	537 (84.3)	Referent
Unknown	12 (1.9)	1.73 (0.88–3.38)
Noninjection drug use within past 12 mo.		
Y	144 (22.6)	1.49 (1.19–1.87)
N	468 (73.5)	Referent
Unknown	25 (3.9)	1.42 (0.87–2.31)
Sputum smear		
Positive	494 (77.5)	2.33 (1.85–2.93)
Negative	112 (17.6)	Referent
Not done	31 (4.9)	1.14 (0.74–1.76)
Cavitary disease‡		
Y	438 (68.8)	2.07 (1.70–2.52)
N	181 (28.4)	Referent
Unknown	18 (2.8)	0.54 (0.33–0.89)
Type of therapy		
Directly observed therapy alone	413 (64.8)	0.62 (0.43–0.90)
Self-administered therapy alone	7 (1.1)	0.38 (0.17–0.88)
Both	173 (27.2)	0.55 (0.37–0.81)
Unknown	44 (6.9)	Referent
Social Vulnerability Index score§		
SES—no health insurance	NA	1.12 (1.04–1.21)

*Outcome of plausible source versus not plausible source for all cases of tuberculosis disease with a genotyped isolate. Excludes cases with missing sex, origin of birth, race/ethnicity, unknown sputum smear status, and children 0–4 years of age. Most likely plausible source identified for each secondary case is included (Figure 1). aOR, adjusted odds ratio; NA, not applicable; SES, socioeconomic status; TB, tuberculosis. †Except for Hispanic or Latino, all are non-Hispanic. ‡Evidence of 1 or more lung cavities with chest radiograph, chest computerized tomography scan, or both. §Social Vulnerability Index includes 16 US Census indicators from the 5-year American Community Survey grouped into 4 themes: SES, household characteristics, racial and ethnic minority status, and housing type and transportation. Ranking values range from 0 to 1, and higher values indicate higher vulnerability and are merged to each case using the patient’s county of residence. ORs based on a 0.2-unit (20%) change for that indicator.

### Machine Learning Predictive Models

After random selection of nonplausible source cases, we ran MLMs under the most likely scenario by using 3,456 observations, among which we used 2,686 (77.8%) for training and 770 (22.2%) for testing (Appendix Table 5). We assessed recall and F1 statistic, which is the harmonic mean of weighted-average recall and weighted-average precision computed as prevalence-weighted averages across the classes. We noted recall and F1 statistic were highest for gradient boosting (recall 0.758; F1 0.752), adaptive boosting (recall 0.751; F1 0.750), and random forest (recall 0.755; F1 0.740) methods; we selected those methods for hyperparameter tuning. The tuned adaptive boosting model had the highest area under the receiver operating characteristic curve (AUC; 0.780) (Appendix). 

**Figure 3:**
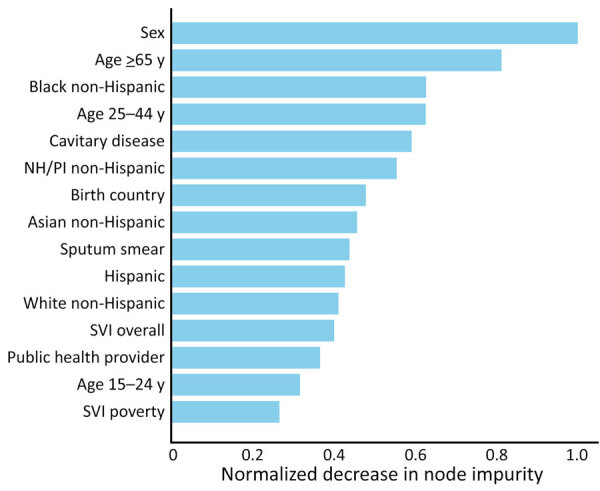
Characteristics from an adaptive boosting model of plausible source cases responsible for recent *Mycobacterium tuberculosis* transmission, United States, 2018–2022. Horizontal bars show the normalized impurity-based importance (mean decrease in node impurity) associated with each predictor; larger values indicate greater importance. NH/PI, Native Hawaiian/Pacific Islander; SVI, Social Vulnerability Index.

We found no reduction in predictive performance for the tuned adaptive boosting model when applied to the test set (F1 0.761; AUC 0.811) versus model training (F1 0.755; AUC 0.780). Sensitivity was 55.4% (95% CI 48.7%–62.2%), specificity 82.7% (95% CI 79.5%–85.8%), and AUC 0.81 (95% CI 0.78–0.84) with the test set. Individual-level factors dominated feature importance and county-level SVI measures were less predictive (Figure 3; Appendix).

### Distribution of Secondary Cases Associated with Plausible Source Cases

The distribution of the number of secondary cases attributed to each plausible source case was right skewed, indicating heterogeneity of TB transmission (Appendix Figure). Most (76.6%, n = 494) plausible source cases transmitted to a single secondary case and comprised 53.6% of all estimated transmission events. Plausible source cases that transmitted to 3–15 secondary cases (8.1%, n = 52) comprised 24.9% of all estimated transmission events.

### Sensitivity Analyses

We restricted analyses to source assignments solely on the basis of wgSNP difference or epidemiologic link and we removed the index of infectiousness from the hierarchy. In both instances, associations for smear positivity and cavitary disease were modestly attenuated but remained statistically significant (Appendix Table 6).

## Discussion

*M. tuberculosis* transmission was rare in the United States during 2018–2022; nonetheless, cases attributed to recent transmission were diagnosed in nearly every US state. In mixed models, sputum smear positivity, cavitary disease, race/ethnicity other than non-Hispanic White or Asian, age 15–44 years, being US-born, and homelessness were associated with being a plausible source for transmission. We found heterogeneous transmission and 8.1% of plausible source cases identified by the recent transmission algorithm were linked to 3–15 secondary cases and accounted for 24.9% of inferred transmission events. Among plausible source cases identified during 2018–2020 that were linked to >3 secondary cases, 86.5% were sputum smear–positive versus 48.1% of all genotyped cases reported during that period; similarly, 76.9% of plausible source cases had cavitary disease versus 36.7% of all genotyped cases. US-born persons only accounted for 27.4% of all genotyped cases, but 71.2% of plausible sources linked to >3 secondary cases were among US-born persons.

Our results align with prior investigations of source-case characteristics. A study from the Netherlands defined source cases as the first case diagnosed in a genotype-matched cluster (*29*). That study found fewer secondary cases for female sex and decreasing numbers of cases with increasing source age. In another study conducted among smear-positive TB patients in Barcelona, Spain, researchers used contact investigations to identify secondary cases and found that more secondary cases occurred after cases in younger adults, those with cavitary disease, and persons who injected drugs (*30*). In Peru, sources identified by using wgSNP distance more often were <34 years of age, were male, and had incarceration history or reported alcohol use or smoking (*31*).

Characteristics of plausible source cases in our study were like characteristics of cases estimated to be attributed to recent transmission. One study found that among the largest 10% of recent transmission clusters, cases attributed to recent transmission were more likely to be in persons who were US-born, American Indian/Alaska Native non-Hispanic, Native Hawaiian/Pacific Islander non-Hispanic, Black non-Hispanic, and Asian non-Hispanic and who reported homelessness (*17*).

In our study, positive sputum smear and cavitary disease, markers of infectiousness and advanced disease (*32**–**34*), were consistently associated with source case status in both GLMM and MLM, including sensitivity analyses where those factors were not used to select between potential source cases. Those associations support the current practice of prioritizing contact investigations for smear-positive and cavitary TB cases (*13*) and suggest that diagnostic delays contribute to transmission. Delays might reflect barriers to care, including homelessness (*4*, *5*,*10*,*35*), or missed diagnoses after seeking healthcare services (*36*). Because TB is uncommon in the United States, clinicians might not routinely consider it in symptomatic patients. Of note, whereas most US TB cases occur among persons born outside the United States, plausible source cases in our study had higher odds of being in US-born persons. Although Asian non-Hispanic persons account for most TB cases (*21*), they were not substantially associated with plausible source status in this study. Missed diagnoses and longer infectious periods among higher-risk groups could explain those findings. Therefore, robust epidemiology and outbreak detection should be used to customize local TB control and prevention efforts focused on at-risk persons.

We found that county-based social determinants of health related to SES (i.e., burdened by housing cost and not having health insurance) were associated with plausible source case attribution in the GLMM (*26*). Those associations could reflect barriers to TB care, including lack of insurance and constrained resources in settings with high housing costs (*37*,*38*). A study that examined data from TB cases reported to the California TB Registry during 2012–2016 found higher TB rates in the lowest SES areas, which defined SES by low education, crowding, poverty, and the California Healthy Places Index (*39*). An ecologic analysis in another study reported that census tracts with lower median incomes, more racial/ethnic minority groups, and more migrants had higher pediatric TB rates; however, overcrowding and unemployment were not associated (*40*). As in those prior studies, our analysis suggests that area-based SES measures could inform TB prevention efforts.

Adaptive boosting ranked male sex and younger age (<65 years) as the main predictors, followed by race/ethnicity, clinical indicators of infectiousness, and birth origin (Figure 3). Ensemble methods outperformed regression-based approaches in this analysis (Appendix Table 5) and have been used to predict TB outcomes, including cluster growth and positive laboratory results (*41*,*42*). The GLMM supports inference through interpretable adjusted associations, and the MLM offers a complementary perspective focused on prediction. Despite differences in ranking, many of the same predictors were influential across both approaches, including markers of infectiousness, age, race/ethnicity, origin of birth, and several social factors. That convergence supports the robustness of our findings.

Strengths of our analysis included the use of wgSNP comparisons to refute case pairs that were not likely to be the result of recent transmission, assessment of both patient- and community-level predictors for association with being a plausible source case, and analysis of plausible source cases of recent transmission at a national level. The first limitation of this analysis is that some cases could have been misattributed as not sources by the algorithm because the source was reported after the secondary case, the source was outside a 10-mile radius, or the secondary case was not genotyped. Although ≈75% of US cases are culture-confirmed (*21*) and >96% of those are genotyped, TB cases in young children, which would predominantly result from recent transmission, have substantially lower rates of culture confirmation. Furthermore, because we limited running the algorithm to starting in 2020, some of the 2018–2019 cases could have been misattributed as not a source if the resulting secondary case was also reported in 2018–2019. We also might have misattributed cases as not sources if all contacts with latent TB infection underwent successful treatment and did not develop TB disease. Second, the COVID-19 pandemic occurred during the analysis period. The pandemic was associated with changes in healthcare seeking behavior that could have affected TB diagnoses; therefore, generalization of our results to other time periods should be done with caution.

In summary, although *M. tuberculosis* transmission is relatively rare in the United States, targeted control efforts could prevent outbreaks that overwhelm public health programs. We identified both patient-level and, to a lesser extent, community-level characteristics as predictors of being a source of recent transmission. Cases with clinical indicators of increased infectiousness were more likely to be transmission sources, supporting current guidance to prioritize those cases for contact investigation. Demographic characteristics associated with being a source case, such as race and origin of birth, differed from those of overall TB cases, highlighting the need for prompt TB diagnosis in US-born persons with risk factors, particularly homelessness and substance use, for preventing outbreaks. In addition, enhanced efforts to promptly diagnose TB in communities with more uninsured residents and those with high housing costs might also reduce transmission. Findings from this analysis suggest that intensifying public health interventions on TB cases in persons with certain demographic and clinical characteristics could yield a greater than expected reduction in *M. tuberculosis* transmission in the United States.

Appendix Additional information on characteristics of plausible source cases responsible for recent *Mycobacterium tuberculosis* transmission, United States, 2018–2022.

## References

[1] World Health Organization. Global tuberculosis report no. 978-92-4-010153-1. Geneva: The Organization; 2024.

[2] Williams PM, Pratt RH, Walker WL, Price SF, Stewart RJ, Feng PI. Tuberculosis—United States, 2023. MMWR Morb Mortal Wkly Rep. 2024;73:265–70. 10.15585/mmwr.mm7312a4 38547024 PMC10986816

[3] LoBue PA, Mermin JH. Latent tuberculosis infection: the final frontier of tuberculosis elimination in the USA. Lancet Infect Dis. 2017;17:e327–33. 10.1016/S1473-3099(17)30248-7 28495525 PMC5614812

[4] Raz KM, Talarico S, Althomsons SP, Kammerer JS, Cowan LS, Haddad MB, et al. Molecular surveillance for large outbreaks of tuberculosis in the United States, 2014–2018. Tuberculosis (Edinb). 2022;136:102232. 10.1016/j.tube.2022.102232 35969928 PMC9530005

[5] Haddad MB, Mitruka K, Oeltmann JE, Johns EB, Navin TR. Characteristics of tuberculosis cases that started outbreaks in the United States, 2002–2011. Emerg Infect Dis. 2015;21:508–10. 10.3201/eid2103.141475 25695665 PMC4344284

[6] Stewart RJ, Raz KM, Burns SP, Kammerer JS, Haddad MB, Silk BJ, et al. Tuberculosis outbreaks in state prisons, United States, 2011–2019. Am J Public Health. 2022;112:1170–9. 10.2105/AJPH.2022.306864 35830666 PMC9342802

[7] Stalter RM, Pecha M, Dov L, Miller D, Ghazal Z, Wortham J, et al. Tuberculosis outbreak in a state prison system—Washington, 2021–2022. MMWR Morb Mortal Wkly Rep. 2023;72:309–12. 10.15585/mmwr.mm7212a3 36952619 PMC10042616

[8] Groenweghe E, Swensson L, Winans KD, Griffin P, Haddad MB, Brostrom RJ, et al. Outbreak of multidrug-resistant tuberculosis—Kansas, 2021–2022. MMWR Morb Mortal Wkly Rep. 2023;72:957–60. 10.15585/mmwr.mm7235a4 37651293

[9] Labuda SM, McDaniel CJ, Talwar A, Braumuller A, Parker S, McGaha S, et al. Tuberculosis outbreak associated with delayed diagnosis and long infectious periods in rural Arkansas, 2010–2018. Public Health Rep. 2022;137:94–101. 10.1177/0033354921999167 33729050 PMC8721759

[10] Mindra G, Wortham JM, Haddad MB, Powell KM. Tuberculosis outbreaks in the United States, 2009–2015. Public Health Rep. 2017;132:157–63. 10.1177/0033354916688270 28147211 PMC5349481

[11] Churchyard G, Kim P, Shah NS, Rustomjee R, Gandhi N, Mathema B, et al. What we know about tuberculosis transmission: an overview. J Infect Dis. 2017;216:S629–35. 10.1093/infdis/jix362 29112747 PMC5791742

[12] Mangione CM, Barry MJ, Nicholson WK, Cabana M, Chelmow D, Coker TR, et al.; US Preventive Services Task Force. Screening for latent tuberculosis infection in adults: US Preventive Services Task Force recommendation statement. JAMA. 2023;329:1487–94. 10.1001/jama.2023.4899 37129649

[13] Cole B, Nilsen DM, Will L, Etkind SC, Burgos M, Chorba T. Essential components of a public health tuberculosis prevention, control, and elimination program: recommendations of the Advisory Council for the Elimination of Tuberculosis and the National Tuberculosis Controllers Association. MMWR Recomm Rep. 2020;69:1–27. 10.15585/mmwr.rr6907a1 32730235 PMC7392523

[14] Smith JP, Cohen T, Dowdy D, Shrestha S, Gandhi NR, Hill AN. Quantifying *Mycobacterium tuberculosis* transmission dynamics across global settings: a systematic analysis. Am J Epidemiol. 2023;192:133–45. 10.1093/aje/kwac181 36227246 PMC10144641

[15] Smith JP, Gandhi NR, Silk BJ, Cohen T, Lopman B, Raz K, et al. A cluster-based method to quantify individual heterogeneity in tuberculosis transmission. Epidemiology. 2022;33:217–27. 10.1097/EDE.0000000000001452 34907974 PMC8886690

[16] Shrestha S, Winglee K, Hill AN, Shaw T, Smith JP, Kammerer JS, et al. Model-based analysis of tuberculosis genotype clusters in the United States reveals high degree of heterogeneity in transmission and state-level differences across California, Florida, New York, and Texas. Clin Infect Dis. 2022;75:1433–41. 10.1093/cid/ciac121 35143641 PMC9412192

[17] Yuen CM, Kammerer JS, Marks K, Navin TR, France AM. Recent transmission of tuberculosis—United States, 2011–2014. PLoS One. 2016;11:e0153728. 10.1371/journal.pone.0153728 27082644 PMC4833321

[18] France AM, Grant J, Kammerer JS, Navin TR. A field-validated approach using surveillance and genotyping data to estimate tuberculosis attributable to recent transmission in the United States. Am J Epidemiol. 2015;182:799–807. 10.1093/aje/kwv121 26464470 PMC5996379

[19] Noppert GA, Yang Z, Clarke P, Davidson P, Ye W, Wilson ML. Contextualizing tuberculosis risk in time and space: comparing time-restricted genotypic case clusters and geospatial clusters to evaluate the relative contribution of recent transmission to incidence of TB using nine years of case data from Michigan, USA. Ann Epidemiol. 2019;40:21–27.e3. 10.1016/j.annepidem.2019.10.001 31711839 PMC6996495

[20] Mamiya H, Schwartzman K, Verma A, Jauvin C, Behr M, Buckeridge D. Towards probabilistic decision support in public health practice: predicting recent transmission of tuberculosis from patient attributes. J Biomed Inform. 2015;53:237–42. 10.1016/j.jbi.2014.11.006 25460204

[21] Centers for Disease Control and Prevention. Reported tuberculosis in the United States, 2021 [cited 2025 Jul 7]. https://www.cdc.gov/tb/statistics/reports/2021/default.htm

[22] Ypma RJ, Altes HK, van Soolingen D, Wallinga J, van Ballegooijen WM. A sign of superspreading in tuberculosis: highly skewed distribution of genotypic cluster sizes. Epidemiology. 2013;24:395–400. 10.1097/EDE.0b013e3182878e19 23446314

[23] Stein RA. Super-spreaders in infectious diseases. Int J Infect Dis. 2011;15:e510–3. 10.1016/j.ijid.2010.06.020 21737332 PMC7110524

[24] Rodriguez CA, Li T, Self JL, Jenkins HE, Horsburgh CR, White LF. Genotyping indicates marked heterogeneity of tuberculosis transmission in the United States, 2009–2018. Epidemiol Infect. 2021;149:e215. 10.1017/S0950268821002041

[25] Melsew YA, Gambhir M, Cheng AC, McBryde ES, Denholm JT, Tay EL, et al. The role of super-spreading events in *Mycobacterium tuberculosis* transmission: evidence from contact tracing. BMC Infect Dis. 2019;19:244. 10.1186/s12879-019-3870-1 30866840 PMC6417041

[26] Centers for Disease Control and Prevention; Agency for Toxic Substances and Disease Registry. CDC/ATSDR Social Vulnerability Index 2022 database [cited 2025 Jul 10]. https://www.atsdr.cdc.gov/placeandhealth/svi/data_documentation_download.html

[27] Walker TM, Ip CL, Harrell RH, Evans JT, Kapatai G, Dedicoat MJ, et al. Whole-genome sequencing to delineate *Mycobacterium tuberculosis* outbreaks: a retrospective observational study. Lancet Infect Dis. 2013;13:137–46. 10.1016/S1473-3099(12)70277-3 23158499 PMC3556524

[28] Chawla NV, Bowyer KW, Hall LO, Kegelmeyer WP. SMOTE: Synthetic Minority Over-sampling Technique. J Artif Intell Res. 2002;16:321–57. 10.1613/jair.953

[29] Borgdorff MW, Nagelkerke NJ, de Haas PE, van Soolingen D. Transmission of *Mycobacterium tuberculosis* depending on the age and sex of source cases. Am J Epidemiol. 2001;154:934–43. 10.1093/aje/154.10.934 11700248

[30] Rodrigo T, Caylà JA, García de Olalla P, Galdós-Tangüis H, Jansà JM, Miranda P, et al. Characteristics of tuberculosis patients who generate secondary cases. Int J Tuberc Lung Dis. 1997;1:352–7. 9432392

[31] Trevisi L, Brooks MB, Becerra MC, Calderón RI, Contreras CC, Galea JT, et al. Who transmits tuberculosis to whom: a cross-sectional analysis of a cohort study in Lima, Peru. Am J Respir Crit Care Med. 2024;210:222–33. 10.1164/rccm.202307-1217OC 38416532 PMC11276835

[32] Lau A, Barrie J, Winter C, Elamy AH, Tyrrell G, Long R. Chest radiographic patterns and the transmission of tuberculosis: implications for automated systems. PLoS One. 2016;11:e0154032. 10.1371/journal.pone.0154032 27105337 PMC4841548

[33] Asadi L, Croxen M, Heffernan C, Dhillon M, Paulsen C, Egedahl ML, et al. How much do smear-negative patients really contribute to tuberculosis transmissions? Re-examining an old question with new tools. EClinicalMedicine. 2022;43:101250. 10.1016/j.eclinm.2021.101250 35036885 PMC8743225

[34] Urbanowski ME, Ordonez AA, Ruiz-Bedoya CA, Jain SK, Bishai WR. Cavitary tuberculosis: the gateway of disease transmission. Lancet Infect Dis. 2020;20:e117–28. 10.1016/S1473-3099(20)30148-1 32482293 PMC7357333

[35] Shrestha S, Cilloni L, Asay GRB, Kammerer JS, Raz K, Shaw T, et al. Model-based analysis of impact, costs, and cost-effectiveness of tuberculosis outbreak investigations, United States. Emerg Infect Dis. 2025;31:497–506. 10.3201/eid3103.240633 40023804 PMC11878319

[36] Wallace RM, Kammerer JS, Iademarco MF, Althomsons SP, Winston CA, Navin TR. Increasing proportions of advanced pulmonary tuberculosis reported in the United States: are delays in diagnosis on the rise? Am J Respir Crit Care Med. 2009;180:1016–22. 10.1164/rccm.200901-0059OC 19679694

[37] Simon AE, Fenelon A, Helms V, Lloyd PC, Rossen LM. HUD housing assistance associated with lower uninsurance rates and unmet medical need. Health Aff (Millwood). 2017;36:1016–23. 10.1377/hlthaff.2016.1152 28583959 PMC5603165

[38] Baker DW, Shapiro MF, Schur CL. Health insurance and access to care for symptomatic conditions. Arch Intern Med. 2000;160:1269–74. 10.1001/archinte.160.9.1269 10809029

[39] Bakhsh Y, Readhead A, Flood J, Barry P. Association of area-based socioeconomic measures with tuberculosis incidence in California. J Immigr Minor Health. 2023;25:643–52. 10.1007/s10903-022-01424-7 36445646 PMC9707420

[40] Myers WP, Westenhouse JL, Flood J, Riley LW. An ecological study of tuberculosis transmission in California. Am J Public Health. 2006;96:685–90. 10.2105/AJPH.2004.048132 16507738 PMC1470555

[41] Althomsons SP, Winglee K, Heilig CM, Talarico S, Silk B, Wortham J, et al. Using machine learning techniques and national tuberculosis surveillance data to predict excess growth in genotyped tuberculosis clusters. Am J Epidemiol. 2022;191:1936–43. 10.1093/aje/kwac117 35780450 PMC10790200

[42] Smith JP, Milligan K, McCarthy KD, Mchembere W, Okeyo E, Musau SK, et al. Machine learning to predict bacteriologic confirmation of *Mycobacterium tuberculosis* in infants and very young children. PLOS Digit Health. 2023;2:e0000249. 10.1371/journal.pdig.0000249 37195976 PMC10191346

