# Supplementary material for: Characteristics of Plausible Source Cases Responsible for Recent Mycobacterium tuberculosis Transmission, United States, 2018–2022
Source: Emerg Infect Dis. 2026 Jun;32(6):880–93. doi: 10.3201/eid3206.260104 (PMC13245205; doi:10.3201/eid3206.260104)
Supplement: Appendix — Additional information on characteristics of plausible source cases responsible for recent Mycobacterium tuberculosis transmission, United States, 2018–2022. [file 26-0104-Techapp-s1.pdf]

Article DOI: https://doi.org/10.3201/eid3206.260104

EID cannot ensure accessibility for supplementary materials supplied by authors. Readers who have difficulty accessing supplementary content should contact the authors for assistance.

Characteristics of Plausible Source Cases Responsible for Recent *Mycobacterium tuberculosis* Transmission, United States, 2018–2022

Appendix

Machine learning (ML) analysis

We compared the performance of 10 ML methods by implementing stratified random sampling to partition the data into training and test sets (75/25). We assessed model performance on the training set using 5-fold cross-validation repeated 10 times. For each repetition, models were trained using 4-folds and validated on the remaining fold, such that each subset was used once for validation. We calculated means and SDs across the 50 validation sets for weighted-average recall, weighted-average precision, F1 statistic, and area under the receiver operating characteristic curve (AUC). Weighted-average recall was calculated as the prevalence-weighted mean of sensitivity and specificity. Weighted-average precision was calculated as the prevalence-weighted mean of positive predictive value and negative predictive value. The F1 statistic is the harmonic mean of weighted-average recall and weighted-average precision (computed as prevalence-weighted averages across the classes) (Appendix Table 5).

Given the imbalance between cases assessed as plausible source versus nonplausible source, we randomly selected cases not estimated to be plausible source cases to achieve a 3:1 ratio of nonplausible to plausible source cases before partitioning the analytic sample into training and test sets (i.e., both sets reflect the 3:1 class ratio). In preliminary analyses, models fit to the full dataset had lower performance. We additionally balanced the two target classes during training using the synthetic minority over-sampling technique (SMOTE); SMOTE was applied to the training data only (i.e., within each cross-validation training fold) (*1*).

We selected the 3 best performing ML methods based on the F1 statistic and tuned each model's hyperparameters using a random search of the hyperparameter space; we generated 50 candidate sets across a range of values for each hyperparameter and compared the sets using 5-fold cross-validation repeated 10 times to determine the best set based on our performance metrics (Appendix Table 5).

We used the independent test set of data not used in model training to evaluate the predictive performance of the best performing tuned model based on the F1 statistic and AUC and to assess model overfitting. We reported AUC, sensitivity, and specificity on the independent test set using a 0.5 probability threshold; we used stratified bootstrapping on the test set with 2,000 resamples to calculate 95% confidence intervals for each metric, defined as the 2.5 and 97.5 percentiles of the resampling distribution.

We reported model findings using scaled relative importance scores in which the feature with the highest variable importance was assigned a score of 1.0. Variable importance scores are interpreted as the importance of each feature in predicting the target, i.e., whether a case is a plausible source or not. Feature importance with adaptive boosting models involved measuring the weighted average of the decrease in node impurity across the boosted trees for each feature.

The 15 most important features included sex, age (aged ≥65 negatively associated), race/ethnicity, origin of birth, and measures of infectiousness (sputum smear positivity and cavitary disease) (Figure 3). Among county-level SVI measures, overall social vulnerability and poverty (one of the SES factors), defined as the percentage of persons living below the federal poverty threshold, were among the top 15 features but were less predictive than individual characteristics; for example, overall vulnerability had ~40% of the relative importance of sex.

Sensitivity analyses

We performed additional analyses to assess the impact of label leakage based on inclusion of infectiousness indicators (i.e., sputum smear positivity and cavitary disease) in the determination of the outcome as well as predictors in the models. The use of the infectiousness index, which includes sputum smear positivity and cavitary disease, was not used to define our outcome directly; we used a hierarchy and only used the index if the most likely plausible source case could not be determined using wgSNP difference or epidemiologic link (12.4% of plausible

source assignments – Appendix Table 4). For the mixed modeling we ran a sensitivity using the subset of cases in which the most likely plausible source case was determined with wgSNP difference or epidemiologic link only (n = 664) and another in which all cases were included (n = 922) but the infectiousness index was not used in the hierarchy (i.e., most likely plausible source cases were determined randomly for 17.2% of the cases versus 4.8% in the base analysis). The difference in the odds ratio point estimate comparing the subset analysis with the base model was −0.18 for sputum smear positivity and −0.13 for cavitary disease (Appendix Table 3).

We re-trained and re-tuned the adaptive boosting machine learning model using the subset of cases in which the most likely plausible source case was determined with wgSNP difference or epidemiologic link only. Performance on the independent test set was comparable to the base model with little difference in discrimination ability (Appendix Table 7).

References

1. Chawla NV, Bowyer KW, Hall LO, Kegelmeyer WP. SMOTE: Synthetic Minority Over-sampling Technique. J Artif Intell Res. 2002;16:321–57. https://doi.org/10.1613/jair.953

Appendix Table 1. Characteristics of all plausible source cases during 2018–2020 responsible for recent *Mycobacterium tuberculosis* transmission during 2020–2022, United States*

Characteristic	Nonplausible source	Plausible source	p value†
Total cases, n = 19,577	18,684 (95.4)	893 (4.6)	
Sex			<0.001
M	11,465 (61.4)	631 (70.7)	
F	7,216 (38.6)	262 (29.3)	
Unknown	3 (0)	0	
Age, y			<0.001
<5	185 (1.0)	0	
5–14	191 (1.0)	13 (1.5)	
15–24	1,802 (9.6)	120 (13.4)	
25–44	5,521 (29.5)	338 (37.9)	
45–64	5,503 (29.5)	337 (37.7)	
≥65	5,482 (29.3)	85 (9.5)	
Origin of birth			<0.001
United States	4,829 (25.8)	536 (60.0)	
Non-US country	13,826 (74.0)	356 (39.9)	
Unknown	29 (0.2)	1 (0.1)	
Race and ethnicity‡			<0.001
Hispanic or Latino	5,459 (29.2)	290 (32.5)	
American Indian/Alaska Native	161 (0.9)	47 (5.3)	
Native Hawaiian/Pacific Islander	215 (1.2)	29 (3.2)	
Black	3,377 (18.1)	325 (36.4)	
Asian	7,161 (38.3)	86 (9.6)	
White	2,115 (11.3)	104 (11.6)	
Multiple race	137 (0.7)	6 (0.7)	
Unknown	59 (0.3)	6 (0.7)	
Resident of a correctional facility at TB diagnosis			<0.001
Y	464 (2.5)	49 (5.5)	
N	18,154 (97.2)	834 (93.4)	
Unknown	66 (0.3)	10 (1.1)	
Experiencing homelessness within past 12 mo.			<0.001
Y	744 (4.0)	138 (15.4)	
N	17,776 (95.1)	740 (82.9)	
Unknown	164 (0.9)	15 (1.7)	
Excess alcohol use within past 12 mo.			<0.001
Y	1,618 (8.7)	184 (20.6)	
N	16,783 (89.8)	687 (76.9)	
Unknown	283 (1.5)	22 (2.5)	
Injection drug use within past 12 mo.			<0.001
Y	208 (1.1)	27 (3.0)	
N	18,004 (96.4)	825 (92.4)	
Unknown	472 (2.5)	41 (4.6)	
Noninjection drug use within past 12 mo.			<0.001
Y	1,213 (6.5)	204 (22.8)	
N	17,022 (91.1)	650 (72.8)	
Unknown	449 (2.4)	39 (4.4)	
Healthcare worker			0.02
Y	652 (3.5)	16 (1.8)	
N	17,248 (92.3)	839 (93.9)	
Unknown	784 (4.2)	38 (4.3)	
Migrant/seasonal worker			0.36
Y	216 (1.2)	15 (1.7)	
N	17,684 (94.6)	840 (94.1)	
Unknown	784 (4.2)	38 (4.3)	
Sputum smear			<0.001
Positive	8,788 (47.0)	621 (69.5)	
Negative	7,759 (41.5)	217 (24.3)	
Not done	2,119 (11.3)	55 (6.2)	
Unknown	18 (0.1)	0	
Cavitary disease§			<0.001
Y	6,638 (35.5)	541 (60.6)	
N	9,877 (52.9)	310 (34.7)	
Unknown	2,169 (11.6)	42 (4.7)	
Previous tuberculosis			0.64
Y	745 (4.0)	37 (4.1)	
N	17,773 (95.1)	851 (95.3)	
Unknown	166 (0.9)	5 (0.6)	

Characteristic	Nonplausible source	Plausible source	p value†
HIV status within past 12 mo.			<0.001
Positive	806 (4.3)	58 (6.5)	
Negative	15,898 (85.1)	801 (89.7)	
Not offered	1,169 (6.3)	19 (2.1)	
Refused	398 (2.1)	5 (0.6)	
Unknown/not reported¶	413 (2.2)	10 (1.1)	
Contact of infectious tuberculosis patient during past 2 years			<0.001
Y	970 (5.2)	122 (13.7)	
Unknown/not reported	17,714 (94.8)	771 (86.3)	
Diabetes mellitus at or before TB diagnosis			0.05
Y	4,327 (23.2)	181 (20.3)	
Unknown/not reported	14,357 (76.8)	712 (79.7)	
Immunosuppression#			0.001
Y	1,174 (6.3)	31 (3.5)	
Unknown/not reported	17,510 (93.7)	862 (96.5)	
End-stage renal disease or chronic renal failure			0.01
Y	576 (3.1)	14 (1.6)	
Unknown/not reported	18,108 (96.9)	879 (98.4)	
Type of therapy			<0.001
Directly observed therapy alone	10,817 (57.9)	592 (66.3)	
Self-administered therapy alone	818 (4.4)	11 (1.2)	
Both	5,906 (31.6)	231 (25.9)	
Unknown	1,143 (6.1)	59 (6.6)	
Median duration of therapy, d (IQR)	247 (185–294)	270 (192–298)	0.001**
Healthcare provider type			0.33
Health department	10,785 (57.7)	528 (59.1)	
Private/other	4,657 (24.9)	200 (22.4)	
Both	1,718 (9.2)	91 (10.2)	
Unknown	1,524 (8.2)	74 (8.3)	
Median Social Vulnerability Index score (IQR)††	0.73 (0.49–0.86)	0.83 (0.66–0.89)	<0.001**
Socioeconomic status	0.65 (0.34–0.86)	0.80 (0.58–0.92)	<0.001**
Household characteristics	0.45 (0.23–0.70)	0.56 (0.32–0.74)	<0.001**
Racial and ethnic minority status	0.91 (0.78–0.96)	0.92 (0.82–0.97)	<0.001**
Housing type and transportation	0.78 (0.61–0.90)	0.83 (0.68–0.91)	<0.001**

*Values are no. (%) except as indicated. TB, tuberculosis.
†χ^2 test or Fisher exact test when <5 cases expected.
‡Except for Hispanic or Latino, all are non-Hispanic.
§Evidence of ≥1 lung cavities with chest radiograph, chest computerized tomography, or both.
¶Includes indeterminate result and test done but result unknown.
#Immunosuppression due to a medical condition, medication or immunosuppressive therapy.
**Wilcoxon rank sum test.
††Overall Social Vulnerability Index includes 16 US Census indicators from the 5-year American Community Survey grouped into 4 themes: socioeconomic status, household characteristics, racial and ethnic minority status, and housing type and transportation. Ranking values range from 0 to 1, and higher values indicate higher vulnerability and are merged to each case using the patient’s county of residence.

Appendix Table 2. Characteristics of most likely plausible source cases during 2018–2020 responsible for recent *Mycobacterium tuberculosis* transmission during 2020–2022, United States*

Characteristic	Nonplausible source	Plausible source	p value†
Total no. cases, (%); n = 19,577	18,932 (96.7)	645 (3.3)	
Sex			<0.001
M	11,640 (61.5)	456 (70.7)	
F	7,289 (38.5)	189 (29.3)	
Unknown	3 (0.0)	0 (0.0)	
Age, y			<0.001
<5	185 (1.0)	0 (0.0)	
5–14	198 (1.0)	6 (0.9)	
15–24	1,828 (9.7)	94 (14.6)	
25–44	5,620 (29.7)	239 (37.1)	
45–64	5,597 (29.6)	243 (37.7)	
≥65	5,504 (29.1)	63 (9.8)	
Origin of birth			<0.001
United States	5,022 (26.5)	343 (53.2)	
Non-US country	13,881 (73.3)	301 (46.7)	
Unknown	29 (0.2)	1 (0.2)	
Race and ethnicity‡			<0.001
Hispanic or Latino	5,513 (29.1)	236 (36.6)	
American Indian/Alaska Native	186 (1.0)	22 (3.4)	
Native Hawaiian/Pacific Islander	221 (1.2)	23 (3.6)	
Black	3,486 (18.4)	216 (33.5)	
Asian	7,175 (37.9)	72 (11.2)	
White	2,150 (11.4)	69 (10.7)	
Multiple race	141 (0.7)	2 (0.3)	
Unknown	60 (0.3)	5 (0.8)	
Resident of a correctional facility at time of TB diagnosis			<0.001
Y	483 (2.6)	30 (4.7)	
N	18,381 (97.1)	607 (94.1)	
Unknown	68 (0.4)	8 (1.2)	
Experiencing homelessness within past 12 mo.			<0.001
Y	793 (4.2)	89 (13.8)	
N	17,973 (94.9)	543 (84.2)	
Unknown	166 (0.9)	13 (2.0)	
Excess alcohol use within past 12 mo.			<0.001
Y	1,683 (8.9)	119 (18.5)	
N	16,961 (89.6)	509 (78.9)	
Unknown	288 (1.5)	17 (2.6)	
Injection drug use within past 12 mo.			<0.001
Y	219 (1.2)	16 (2.5)	
N	18,229 (96.3)	600 (93.0)	
Unknown	484 (2.6)	29 (4.5)	
Noninjection drug use within past 12 mo.			<0.001
Y	1,273 (6.7)	144 (22.3)	
N	17,199 (90.9)	473 (73.3)	
Unknown	460 (2.4)	28 (4.3)	
Healthcare worker			0.11
Y	655 (3.5)	13 (2.0)	
N	17,486 (92.4)	601 (93.2)	
Unknown	791 (4.2)	31 (4.8)	
Migrant/seasonal worker			0.04
Y	217 (1.1)	14 (2.2)	
N	17,924 (94.7)	600 (93.0)	
Unknown	791 (4.2)	31 (4.8)	
Sputum smear			<0.001
Positive	8,909 (47.1)	500 (77.5)	
Negative	7,863 (41.5)	113 (17.5)	
Not done	2,142 (11.3)	32 (5.0)	
Unknown	18 (0.1)	0 (0.0)	
Cavitary disease§			<0.001
Y	6,737 (35.6)	442 (68.5)	
N	10,004 (52.8)	183 (28.4)	
Unknown	2,191 (11.6)	20 (3.1)	
Previous tuberculosis			0.86
Y	754 (4.0)	28 (4.3)	
N	18,012 (95.1)	612 (94.9)	
Unknown	166 (0.9)	5 (0.8)	

Characteristic	Nonplausible source	Plausible source	p value†
HIV status during past 12 mo.			<0.001
Positive	829 (4.4)	35 (5.4)	
Negative	16,115 (85.1)	584 (90.5)	
Not offered	1,174 (6.2)	14 (2.2)	
Refused	399 (2.1)	4 (0.6)	
Unknown/not reported¶	415 (2.2)	8 (1.2)	
Contact of infectious tuberculosis patient during prior 2 y			<0.001
Y	1,030 (5.4)	62 (9.6)	
Unknown/not reported	17,902 (94.6)	583 (90.4)	
Diabetes mellitus at or before TB diagnosis			0.47
Y	4,367 (23.1)	141 (21.9)	
Unknown/not reported	14,565 (76.9)	504 (78.1)	
Immunosuppression#			0.009
Y	1,181 (6.2)	24 (3.7)	
Unknown/not reported	17,751 (93.8)	621 (96.3)	
End-stage renal disease or chronic renal failure			0.05
Y	579 (3.1)	11 (1.7)	
Unknown/not reported	18,353 (96.9)	634 (98.3)	
Type of therapy			<0.001
Directly observed therapy alone	10,993 (58.1)	416 (64.5)	
Self-administered therapy alone	820 (4.3)	9 (1.4)	
Both	5,963 (31.5)	174 (27.0)	
Unknown	1,156 (6.1)	46 (7.1)	
Median duration of therapy, d (IQR)	247 (185–294)	273 (193–299)	<0.001**
Healthcare provider type			0.45
Health department	10,930 (57.7)	383 (59.4)	
Private/other	4,714 (24.9)	143 (22.2)	
Both	1,747 (9.2)	62 (9.6)	
Unknown	1,541 (8.1)	57 (8.8)	
Median Social Vulnerability Index score (IQR)††	0.73 (0.49–0.86)	0.80 (0.65–0.89)	<0.001**
Socioeconomic status	0.65 (0.34–0.87)	0.79 (0.56–0.90)	<0.001**
Household characteristics	0.46 (0.23–0.70)	0.55 (0.32–0.74)	<0.001**
Racial and ethnic minority status	0.91 (0.78–0.97)	0.91 (0.81–0.97)	0.006**
Housing type and transportation	0.78 (0.61–0.90)	0.82 (0.67–0.91)	<0.001**

*Values are no. (%) except as indicated. TB, tuberculosis.
†χ^2 test or Fisher exact test when <5 cases expected.
‡Except for Hispanic or Latino, all are non-Hispanic.
§Evidence of $\geq$1 lung cavities with chest radiograph, chest computerized tomography, or both.
¶Includes indeterminate result and test done but result unknown.
#Immunosuppression due to a medical condition, medication or immunosuppressive therapy.
**Wilcoxon rank sum test.
††Overall Social Vulnerability Index includes 16 US Census indicators from the 5-year American Community Survey grouped into 4 themes: socioeconomic status, household characteristics, racial and ethnic minority status, and housing type and transportation. Ranking values range from 0 to 1, and higher values indicate higher vulnerability and are merged to each case using the patient's county of residence.

Appendix Table 3. Characteristics of most likely plausible source cases during 2018–2020 responsible for recent *Mycobacterium tuberculosis* transmission during 2020–2022, United States*

Characteristic	Nonplausible source	Plausible source for ≥1 TB cases		p value†
		1–2 cases	≥3 cases	
Total no. cases, (%); n = 19,577	18,932 (96.7)	593 (3.0)	52 (0.3)	
Sex				<0.001
M	11,640 (61.5)	415 (70.0)	41 (78.9)	
F	7,289 (38.5)	178 (30.0)	11 (21.1)	
Unknown	3 (0.0)	0 (0.0)	0 (0.0)	
Age, y				<0.001
<5	189 (1.0)	0 (0.0)	0 (0.0)	
5–14	198 (1.0)	6 (1.0)	0 (0.0)	
15–24	1,828 (9.7)	87 (14.7)	7 (13.5)	
25–44	5,620 (29.7)	214 (36.1)	25 (48.1)	
45–64	5,597 (29.6)	225 (37.9)	18 (34.6)	
≥65	5,504 (29.1)	61 (10.3)	2 (3.8)	
Origin of birth				<0.001
United States	5,022 (26.5)	306 (51.6)	37 (71.2)	
Non-US country	13,881 (73.3)	286 (48.2)	15 (28.8)	
Unknown	29 (0.2)	1 (0.2)	0 (0.0)	
Race and ethnicity‡				<0.001
Hispanic or Latino	5,513 (29.1)	227 (38.3)	9 (17.3)	
American Indian/Alaska Native	186 (1.0)	15 (2.5)	7 (13.5)	
Native Hawaiian/Pacific Islander	221 (1.2)	22 (3.7)	1 (1.9)	
Black	3,486 (18.4)	188 (31.7)	28 (53.8)	
Asian	7,175 (37.9)	68 (11.5)	4 (7.7)	
White	2,150 (11.4)	66 (11.1)	3 (5.8)	
Multiple race	141 (0.7)	2 (0.3)	0 (0.0)	
Unknown	60 (0.3)	5 (0.8)	0 (0.0)	
Resident of a correctional facility at time of TB diagnosis				<0.001
Y	483 (2.5)	27 (4.5)	3 (5.8)	
N	18,381 (97.1)	559 (94.3)	48 (92.3)	
Unknown	68 (0.4)	7 (1.2)	1 (1.9)	
Experiencing homelessness within past 12 mo.				<0.001
Y	793 (4.2)	78 (13.1)	11 (21.1)	
N	17,973 (94.9)	504 (85.0)	39 (75.0)	
Unknown	166 (0.9)	11 (1.9)	2 (3.9)	
Excess alcohol use within past 12 mo.				<0.001
Y	1,683 (8.7)	109 (18.4)	10 (19.2)	
N	16,961 (89.6)	471 (79.4)	38 (73.1)	
Unknown	288 (1.5)	13 (2.2)	4 (7.7)	
Injection drug use within past 12 mo.				<0.001
Y	219 (1.2)	16 (2.7)	0 (0.0)	
N	18,229 (96.3)	553 (93.2)	47 (90.4)	
Unknown	472 (2.5)	24 (4.1)	5 (9.6)	
Noninjection drug use within past 12 mo.				<0.001
Y	1,273 (6.7)	124 (20.9)	20 (38.5)	
N	17,199 (90.9)	446 (75.2)	27 (51.9)	
Unknown	460 (2.4)	23 (3.9)	5 (9.6)	
Healthcare worker				0.27
Y	665 (3.5)	13 (2.2)	0 (0.0)	
N	17,486 (92.4)	551 (92.9)	50 (96.2)	
Unknown	791 (4.2)	29 (4.9)	2 (3.8)	
Migrant/seasonal worker				0.09
Y	217 (1.2)	14 (2.4)	0 (0.0)	
N	17,924 (94.7)	550 (92.7)	50 (96.2)	
Unknown	791 (4.2)	29 (4.9)	2 (3.8)	
Sputum smear				<0.001
Positive	8,909 (47.1)	455 (76.7)	45 (86.5)	
Negative	7,863 (41.5)	108 (18.2)	5 (9.6)	
Not done	2,142 (11.3)	30 (5.1)	2 (3.9)	
Unknown	18 (0.1)	0 (0.0)	0 (0.0)	
Cavitary disease§				<0.001
Y	6,737 (35.6)	402 (67.8)	40 (76.9)	
N	10,004 (52.8)	173 (29.2)	10 (19.2)	
Unknown	2,191 (11.6)	18 (3.0)	2 (3.9)	
Previous tuberculosis				0.88
Y	754 (4.0)	25 (4.2)	3 (5.8)	

Characteristic	Nonplausible source	Plausible source for >1 TB cases		p value†
		1–2 cases	>3 cases	
N	18,012 (95.1)	563 (94.9)	49 (94.2)	
Unknown	166 (0.9)	5 (0.9)	0 (0.0)	
HIV status during past 12 mo.				<0.001
Positive	829 (4.4)	32 (5.4)	3 (5.8)	
Negative	16,115 (85.1)	536 (90.4)	48 (92.3)	
Not offered	1,174 (6.2)	13 (2.2)	1 (1.9)	
Refused	399 (2.1)	4 (0.7)	0 (0.0)	
Unknown/not reported¶	415 (2.2)	8 (1.4)	0 (0.0)	
Contact of infectious tuberculosis patient during prior 2 y				<0.001
Y	1030 (5.4)	55 (9.3)	7 (13.5)	
Unknown/not reported	17,902 (94.6)	538 (90.7)	45 (86.5)	
Diabetes mellitus at or before TB diagnosis				0.14
Y	4,367 (23.1)	135 (22.8)	6 (11.5)	
Unknown/not reported	14,565 (76.9)	458 (77.2)	46 (88.5)	
Immunosuppression#				0.03
Y	1,181 (6.2)	22 (3.7)	2 (3.8)	
Unknown/not reported	17,751 (93.8)	571 (96.3)	50 (96.2)	
End-stage renal disease or chronic renal failure				0.13
Y	579 (3.1)	10 (1.7)	1 (1.9)	
Unknown/not reported	18,353 (96.9)	583 (98.3)	51 (98.1)	
Type of therapy				<0.001
Directly observed therapy alone	10,993 (58.1)	384 (64.8)	32 (61.5)	
Self-administered therapy alone	820 (4.3)	9 (1.5)	0 (0.0)	
Both	5,963 (31.5)	156 (26.3)	18 (34.6)	
Unknown	1,156 (6.1)	44 (7.4)	2 (3.9)	
Median duration of therapy, d (IQR)	247 (185–294)	273 (192–298)	274 (232–300)	<0.001**
Healthcare provider type				0.09
Health department	10,930 (57.7)	355 (59.9)	28 (53.8)	
Private/other	4,714 (24.9)	132 (22.3)	11 (21.2)	
Both	1,747 (9.2)	51 (8.6)	11 (21.2)	
Unknown	1,541 (8.2)	55 (9.3)	2 (3.8)	
Median Social Vulnerability Index score (IQR)††	0.73 (0.49–0.86)	0.80 (0.64–0.89)	0.86 (0.78–0.89)	<0.001**
Socioeconomic status	0.65 (0.34–0.86)	0.77 (0.56–0.88)	0.84 (0.70–0.92)	<0.001**
Household characteristics	0.46 (0.23–0.70)	0.54 (0.31–0.74)	0.57 (0.32–0.77)	<0.001**
Racial and ethnic minority status	0.91 (0.78–0.97)	0.91 (0.81–0.97)	0.93 (0.83–0.97)	0.01**
Housing type and transportation	0.78 (0.61–0.90)	0.82 (0.67–0.91)	0.85 (0.68–0.91)	<0.001**

*Values are no. (%) except as indicated. TB, tuberculosis.
†χ^2 test or Fisher exact test when <5 cases expected; indicates differences across the 3 categories.
‡Except for Hispanic or Latino, all are non-Hispanic.
§Evidence of >1 lung cavities with chest radiograph, chest computerized tomography, or both.
¶Includes indeterminate result and test done but result unknown.
#Immunosuppression due to a medical condition, medication or immunosuppressive therapy.
**Kruskal-Wallis test.
††Overall Social Vulnerability Index includes 16 US Census indicators from the 5-year American Community Survey grouped into 4 themes: socioeconomic status, household characteristics, racial and ethnic minority status, and housing type and transportation. Ranking values range from 0 to 1, and higher values indicate higher vulnerability and are merged to each case using the patient's county of residence.

Appendix Table 4. Method of selection for most likely plausible source case identified during 2018–2020 for secondary cases attributed to recent transmission of tuberculosis during 2020–2022

Selection method, n = 922	No. (%)
Fewest wgSNP difference	753 (81.7)
Epidemiologic link	11 (1.2)
Highest index of infectiousness	114 (12.4)
Random	44 (4.8)

Appendix Table 5. Performance metrics of machine learning (ML) models for prediction of most likely plausible source case identified during 2018–2020 for secondary cases attributed to recent transmission of tuberculosis during 2020–2022*

ML method	Weighted-average recall	Weighted-average precision	F1	AUC†
Gradient Boosting	0.758 (0.019)	0.750 (0.022)	0.752 (0.021)	0.774 (0.027)
Adaptive Boosting	0.751 (0.020)	0.751 (0.023)	0.750 (0.021)	0.766 (0.028)
Random Forest	0.755 (0.018)	0.736 (0.021)	0.740 (0.022)	0.756 (0.026)
Logistic Regression	0.712 (0.020)	0.771 (0.021)	0.728 (0.019)	0.781 (0.024)
Ridge Regression	0.706 (0.019)	0.774 (0.022)	0.724 (0.018)	0.781 (0.025)
Decision Tree	0.689 (0.021)	0.697 (0.024)	0.692 (0.021)	0.598 (0.023)
Artificial Neural Network	0.699 (0.035)	0.753 (0.029)	0.713 (0.029)	0.743 (0.032)
Naïve Bayes	0.582 (0.034)	0.746 (0.032)	0.607 (0.034)	0.715 (0.031)
Support Vector Machine – radial kernel	0.650 (0.021)	0.714 (0.027)	0.670 (0.021)	0.684 (0.028)
Support Vector Machine – linear kernel	0.604 (0.180)	0.751 (0.055)	0.572 (0.206)	0.732 (0.058)
Tuned models				
Gradient Boosting (tuned)	0.758 (0.021)	0.756 (0.022)	0.756 (0.021)	0.779 (0.026)
Adaptive Boosting (tuned)	0.751 (0.021)	0.762 (0.021)	0.755 (0.020)	0.780 (0.026)
Random Forest (tuned)	0.759 (0.020)	0.754 (0.022)	0.755 (0.021)	0.775 (0.027)

*Results are reported as mean (SD) across validation resamples using the training data with 5-fold cross-validation repeated 10 times. Tuned models used random search over 50 candidate hyperparameter sets. Weighted-average recall and weighted-average precision were calculated as prevalence-weighted averages across the plausible source and nonplausible source classes.
†AUC – area under the receiver operating characteristic curve

Appendix Table 6. Sensitivity results for multivariable models: plausible source cases identified during 2018–2020 for secondary cases attributed to recent transmission of tuberculosis during 2020–2022

Analysis	Sputum smear positivity, aOR (95% CI)	Cavitary disease, aOR (95% CI)
All plausible sources	1.71 (1.42–2.07)	1.69 (1.42–2.00)
Most likely – base model	2.33 (1.95–2.93)	2.07 (1.70–2.52)
Most likely – wgSNP or epidemiologic link subset*	2.15 (1.71–2.72)	1.94 (1.59–2.38)
Most likely – index of infectiousness omitted†	2.03 (1.63–2.53)	1.89 (1.56–2.30)

*Analysis included the subset of cases in which the most likely plausible source case was determined using wgSNP analysis or epidemiologic link (n = 664).
†Infectiousness indicators (sputum smear positivity and cavitary disease) were not used to determine the most likely plausible source case (n = 922).

Appendix Table 7. Performance metrics for adaptive boosting machine learning model: most likely plausible source cases identified during 2018–2020 for secondary cases attributed to recent transmission of tuberculosis during 2020–2022*

Analysis	Sensitivity (95% CI)	Specificity (95% CI)	AUC (95% CI)
Most likely – base model	55.4 (48.7–62.2)	82.7 (79.5–85.8)	0.81 (0.78–0.84)
Most likely – wgSNP or epidemiologic link subset†	54.0 (46.6–61.4)	84.1 (81.2–87.0)	0.79 (0.76–0.83)

*95% CI calculated using stratified bootstrapping with 2,000 resamples, defined as the 2.5 and 97.5 percentiles of the resampling distribution. AUC, area under the receiver operating characteristic curve.
†Model training and testing included the subset of cases in which the most likely plausible source case was determined using wgSNP analysis or epidemiologic link.

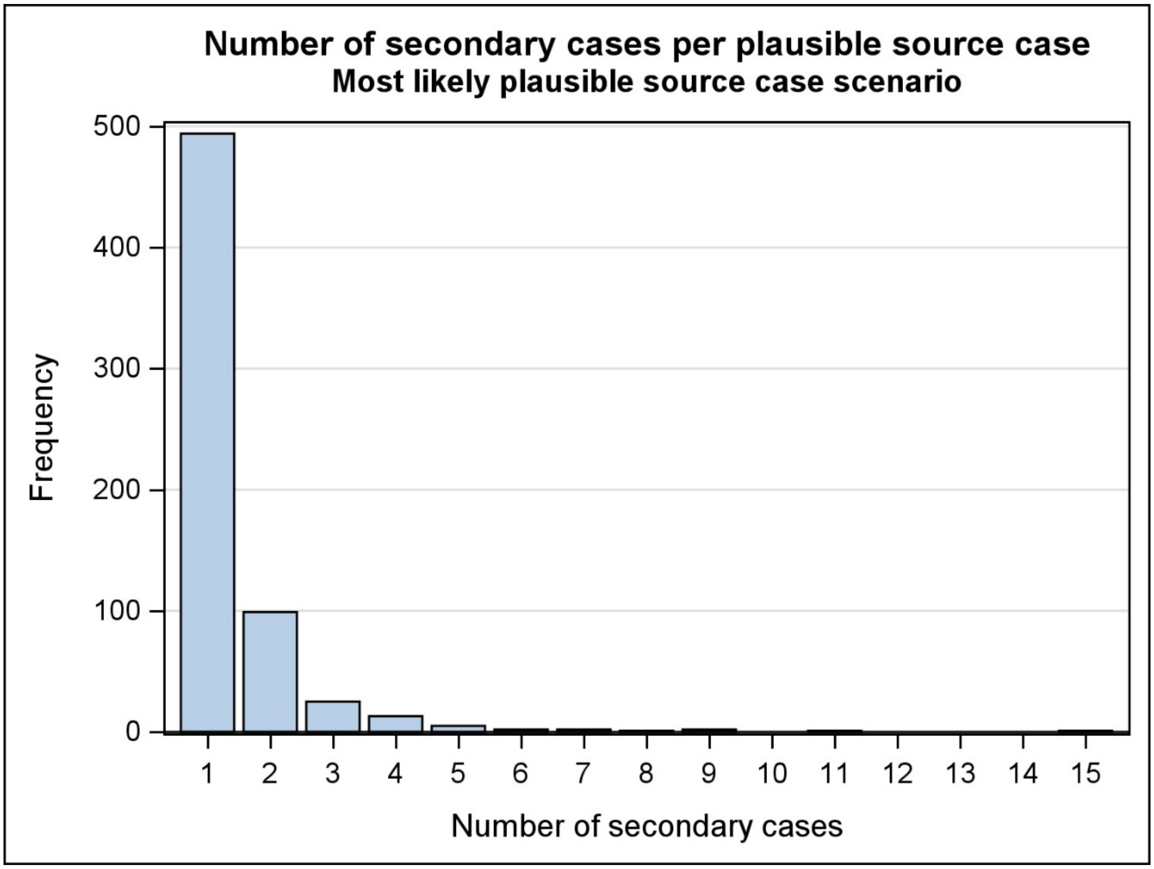

Appendix Figure. Distribution of the number of secondary cases attributed to recent transmission during 2020–2022 for plausible source cases identified during 2018–2020.
